# Updated insights into adverse events associated with mepolizumab: a disproportionality analysis from the FDA adverse event reporting system database

**DOI:** 10.3389/fmed.2024.1449194

**Published:** 2024-10-25

**Authors:** Shan Lin, Dachen Luo, Zonglian Gong, Qingyuan Zhan

**Affiliations:** ^1^Department of Respiratory and Critical Care Medicine, Affiliated Hospital of North Sichuan Medical College, Nanchong, Sichuan, China; ^2^Department of Pulmonary and Critical Care Medicine, Center of Respiratory Medicine, National Center for Respiratory Medicine, China-Japan Friendship Hospital, Beijing, China

**Keywords:** mepolizumab, asthma, adverse events, pharmacovigilance, FAERS

## Abstract

**Background:**

Mepolizumab, a monoclonal antibody targeting interleukin-5, is used to treat severe eosinophilic asthma and other eosinophilia-related conditions. Given its growing use, there is a pressing need for the latest data to improve the understanding and management of its adverse events (AEs). This study aimed to investigate the safety of mepolizumab by analyzing the pharmacovigilance database of the US Food and Drug Administration.

**Methods:**

The AE signals associated with mepolizumab from 2015 to 2024 were analyzed and the correlations using reporting ratios (RORs) quantified. Subgroup analyses were conducted to understand AEs in individuals ≤ 18 years of age. We also used time-to-onset (TTO) analysis to examine AE occurrence patterns.

**Results:**

In total, 82,478 AE reports linked to mepolizumab therapy were included. Our analysis, involving 24,156 patients, revealed a predominance of female patients, with the highest incidence of AEs occurring in those aged 18–65 years. Disproportionality analyses revealed significant signals across various system organ classifications (SOCs), most prominently respiratory, thoracic, and mediastinal disorders (ROR = 5.12, 95% confidence intervals [CI] 5.03–5.21), infections and infestations (ROR = 1.86, 95% CI 1.81–1.90), and immune system disorders (ROR = 1.14, 95% CI 1.08–1.21). The highest ROR was found for asthma crisis (ROR = 104.90, 95% CI 95.31–115.44) at the preferred term (PT) level, and the other notables were coronavirus infection (ROR = 7.33, 95% CI 6.05–8.88) and coronavirus disease 2019 (COVID-19) (ROR = 1.34, 95% CI 1.23–1.47). A subgroup analysis of patients ≤ 18 years old identified four significant SOC signals, with the highest ROR in respiratory, thoracic, and mediastinal disorders (ROR = 5.28, 95% CI 4.17–6.68). PT analysis revealed significant AEs, such as wheezing, bronchospasm, and chest discomfort. TTO analysis revealed that 18.5% of AEs occurred within the first 30 days of treatment. The Weibull shape parameter indicated an “early failure-type” pattern for mepolizumab-associated AEs, underscoring the need for vigilant monitoring during the initial stages of therapy.

**Conclusion:**

Our study highlights the importance of post-market surveillance for monitoring the safety of mepolizumab, which revealed significant AE signals, particularly for respiratory diseases, infections, and immune system complications. The association with opportunistic infections, including COVID-19, highlights the need for vigilant surveillance and further research.

## Introduction

Asthma is a prevalent chronic inflammatory respiratory condition characterized by airway hyperreactivity, inflammation, and reversible obstruction (1). Clinically, it manifests with diverse symptoms, including recurrent wheezing, coughing, chest tightness, and breathing difficulties that intensify at night or early in the morning. However, severe asthma episodes can escalate to acute respiratory failure. The World Health Organization estimates that approximately 300 million individuals are affected by asthma globally, although its prevalence varies across regions and countries (2). The incidence of asthma is influenced by genetic factors; environmental conditions such as air pollution and allergen exposure; and lifestyle choices, including smoking and diet (3, 4).

Asthma pathogenesis involves a complex interplay between genetic predispositions, environmental factors, and the immune system. Asthma develops as a chronic inflammatory response in the airways that leads to increased airway responsiveness, structural remodeling, and symptom manifestation (5). Several cytokines and chemokines play pivotal roles in this inflammatory response. Of these, interleukin-5 (IL-5) produced primarily by Th2 type T cells is critical for the growth, differentiation, recruitment, and activation of eosinophils, which are key effector cells in the inflammatory process (6). Eosinophils release various mediators contributing to airway damage and are a key factor in symptomatology. Elevated IL-5 levels are closely linked to the pathological processes in asthma, particularly in eosinophilic phenotypes of the disease (7). Thus, targeting IL-5 with monoclonal antibodies, such as mepolizumab, has proven to be an effective for severe eosinophilic asthma treatment. Approved by the U.S. Food and Drug Administration (FDA) in 2015, mepolizumab reduces eosinophil-mediated inflammation and tissue damage by inhibiting the biological activities of eosinophils and reducing their levels. It is indicated for the treatment of severe eosinophilic asthma, eosinophilic granulomatosis with polyangiitis, hypereosinophilic syndrome, and chronic rhinosinusitis with nasal polyps (8, 9).

Despite the established clinical efficacy, tolerability, and safety of mepolizumab, which has been demonstrated in controlled trials and real-world studies, common adverse effects such as headaches and back pain are frequently reported (10–12). Additionally, severe adverse events (AEs) have been documented, including the exacerbation of symptoms associated with hypereosinophilic syndrome, infections caused by *Mycobacterium abscessus*, eosinophilic gastroenteritis, and peripheral T-cell lymphoma (13). Furthermore, existing studies on mepolizumab-related AEs have relied on outdated data, which undermines their relevance and applicability to the drug's current clinical profile (14, 15). The dynamic nature of drug safety reports and the evolving clinical use of mepolizumab necessitates continuous updates to ensure that safety assessments accurately reflect the latest data. This pressing need underscores the significance of this research for the development of more effective and safer therapeutic strategies for managing mepolizumab-related AEs. The U.S. Food and Drug Administration Adverse Event Reporting System (FAERS) database is the largest publicly accessible pharmacovigilance database. This database compiles reports of drug-related adverse events from both domestic and international sources (16). This study aimed to evaluate the AEs associated with mepolizumab by analyzing post-marketing data, thereby providing valuable insights for ongoing clinical monitoring and identifying potential risks associated with mepolizumab therapy.

## Methods

### Guideline

This pharmacovigilance disproportionality analysis has been prepared in accordance with the latest Reporting of A Disproportionality Analysis for Drug Safety Signal Detection Using Individual Case Safety Reports in Pharmacovigilance (READUS-PV) guidelines (17). These guidelines are designed to enhance the transparency, completeness, and accuracy of reporting, ensuring proper interpretation and evidence-based decision-making in drug safety.

### Study design and data sources

In this study, we performed a disproportionality analysis using FAERS database to explore the association between mepolizumab and its AEs. Our methodology involved a comparative analysis of the incidence rates of AEs associated with mepolizumab with those associated with all other drugs recorded in the FAERS database. The data for this study were sourced from the publicly available FAERS quarterly data extraction files accessible through the FDA website. To align with the FDA-approved administration schedule for mepolizumab, we included all relevant reports from the FAERS database spanning from the fourth quarter of 2015 to the first quarter of 2024, thereby providing a robust and extensive dataset for our analysis.

### Data extraction and descriptive analysis

**Figure 1:**
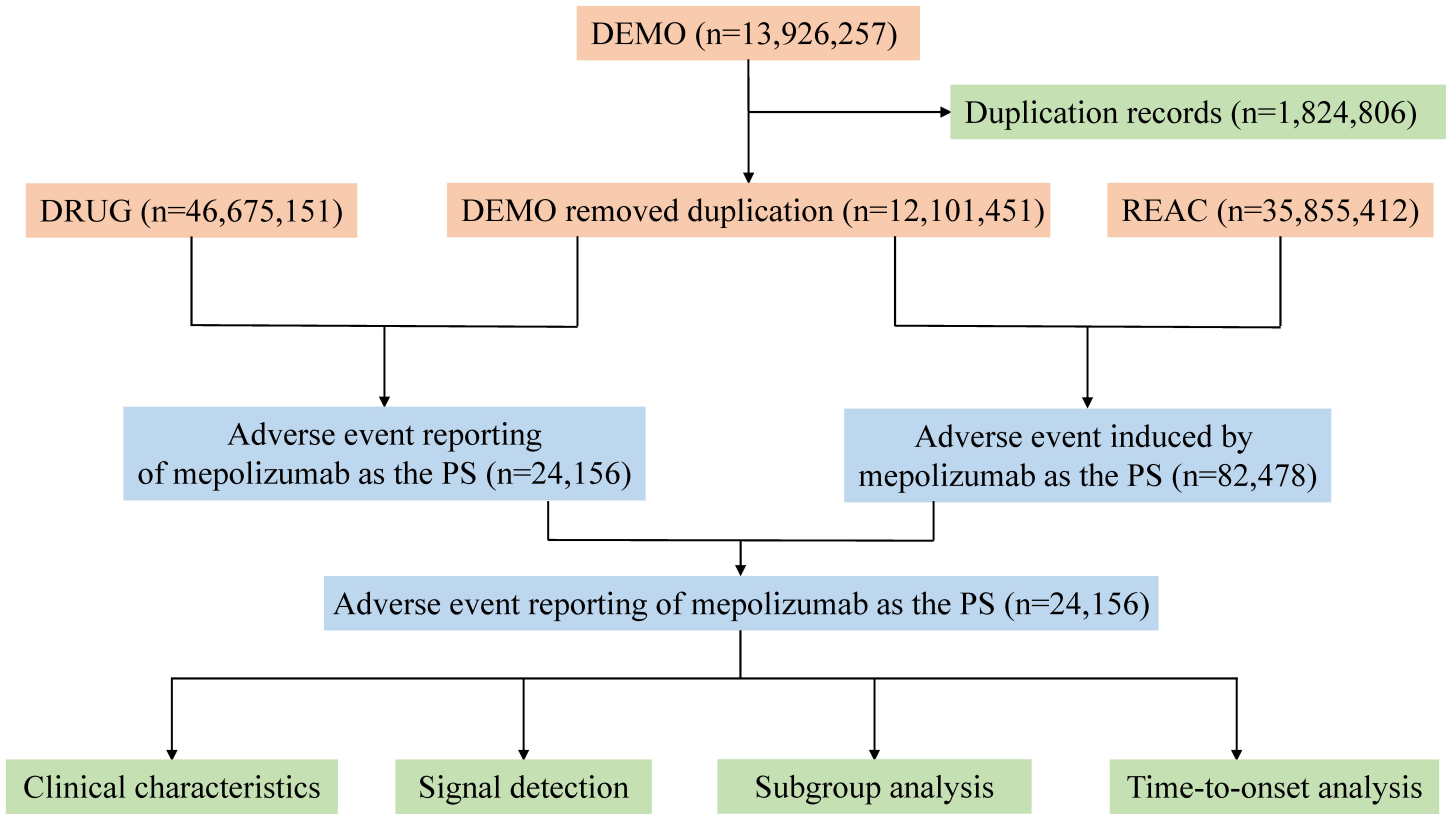
The process of selecting mepolizumab-associated AEs from FAERS database. FDA, Food and Drug Administration; AEs, adverse events; FAERS, FDA Adverse Event Reporting System.

The FAERS database has been meticulously structured into seven principal data files: demographic information (DEMO), drug details (DRUG), adverse event descriptions (REAC), patient outcomes (OUTC), reporting sources (RPSR), medication dates (THER), and indications for medication use (INDI). In addition, a separate file is maintained for entries deleted by the US FDA or the manufacturer for reasons such as duplications or mergers. For our analysis, all data were imported into R software version 4.2.2, and a rigorous deduplication process was implemented prior to statistical analysis. Master IDs were used to link datasets, and case IDs were used as primary filters to eliminate duplicates. To identify relevant cases, both the common name (mepolizumab) and brand name (Nucala) were used in the DRUG file, with the role_code field used to identify drugs classified as primary suspects. A manual review process was crucial when selecting records for inclusion in the study, especially when duplicate case IDs were identified; in such cases, records with the highest primary ID was retained. AEs reported in FAERS were coded using the preferred terminology (PT) from the Medical Dictionary for Regulatory Activities (MedDRA), organized into 27 system organ categories (SOCs). Owing to the nuanced structure of MedDRA, where one PT may correspond to multiple SOCs, MedDRA version 26.0 was used to ensure accurate categorization of AEs at the precise SOC level in R. Wherever possible, a detailed description of the clinical characteristics associated with each report has been provided, including variables such as sex, age, weight, reporting region, drug indication, outcome, and the identity of the reporter. However, the total number of outcomes may exceed the total number of reports owing to certain entries documenting multiple outcomes. Figure 1 illustrates a detailed flowchart outlining the comprehensive process of data extraction, deduplication, and analysis and provides a clear and structured overview of the methodology used in our study.

### Statistical analysis

Our analysis is underpinned by the reporting odds ratio (ROR) algorithm, which is crucial for synthesizing data based on a structured 2 × 2 table. This algorithm is a part of a comprehensive analytical framework that incorporates the Proportional Reporting Ratio (PRR) and Bayesian methods (Empirical Bayesian Geometric Mean [EBGM]). Our study specifically examined the signal strength of mepolizumab-related reports in the FAERS database, focusing on its association with AEs across all SOC levels. A significant aspect of our methodology involved identifying positive signals, recognized when the lower limit of the 95% confidence interval (CI) for the ROR exceeded a threshold value of 1. This threshold indicates a statistically significant increased likelihood of AEs compared to other drugs within the same database, suggesting potential safety signals that warrant further investigation.

### Time-to-onset analysis

In our study, the time to onset (TTO) was meticulously defined as the interval from the initiation of mepolizumab treatment, noted as START_DT in the THER file, to the occurrence of an AE, marked by EVENT_DT in the DEMO file. To maintain the integrity of our TTO analysis, reports compromised by data entry errors such as AEs recorded before the treatment start date, inaccurate date entries, or missing information were excluded.

TTO analysis included examining the medians and quartiles to provide a comprehensive overview of the data distribution. In addition, we employed the Weibull shape parameter (WSP) test to evaluate the changes in the incidence of AEs over time. This assessment is pivotal for understanding the risk dynamics associated with mepolizumab use. The Weibull distribution, forms the basis of this test and is characterized by two main parameters: the scale (α) and shape (β), which determine whether the likelihood of experiencing an AE increases or decreases over time.

The methodology and selection criteria for these specific parameters were based on insights from previous studies (18, 19). WSP tests were performed using the R statistical software, ensuring a thorough and reliable analysis of the TTOs associated with mepolizumab use, thereby providing vital insights into its safety profile over time.

## Results

### Descriptive analysis

We carefully curated data from the FAERS database and compiled 82,478 AE reports after diligently eliminating duplicates. The detailed clinical characteristics in these cases are described in Table 1. Our demographic analysis, encompassing 24,156 patients, showed a predominance of females (11, 136) compared to males (5, 174). The highest incidence of AEs was noted in the 18–65 age group, representing 17.1% of the cases (*n* = 4,127). Regarding the sources of AE reports, healthcare professionals were the primary reporters at 31.45% (*n* = 7,598), whereas consumer reports comprised a substantial 67.28% (*n* = 16,253). Geographically, 55.8% of the AE reports originated in the United States, with the remaining 44.1% originating from non-U.S. regions, with hospitalization being the most frequent outcome among these AEs, accounting for 21.0% of the cases (*n* = 6,034). Notably, a significant proportion of AEs occurred within the first 30 days after dosing (18.5%, *n* = 708), with additional occurrences reported beyond 360 days (43.6%, *n* = 1,669). These findings highlight the critical-risk periods for patients treated with mepolizumab and underscore the need for heightened vigilance during these specific intervals.

**Table 1:** Clinical characteristics of patients with mepolizumab-related AEs.

**Characteristics**	**Case number**	**Case proportion, %**
**Gender**		
Female	11,136	0.461
Male	5,174	0.214
Not Specified	7,846	0.325
**Age (Years)**		
< 18	161	0.007
≥18, < 65	4,127	0.171
≥65, < 85	2,615	0.108
≥85	85	0.004
Not Specified	17,168	0.711
**Weight**		
< 50 kg	72	0.003
50–100 kg	956	0.04
>100 kg	233	0.01
Not Specified	22,895	0.948
**Reporters**		
Health-professional	16,253	0.6728
Consumer	7,598	0.3145
Not specified	305	0.0126
**Report countries**		
US	13,476	0.558
Non-US	10,646	0.441
Not specified	34	0.001
**Reporting year**		
2015	3	0.0001
2016	409	0.0169
2017	1,181	0.0489
2018	2,268	0.0939
2019	3,213	0.133
2020	2,868	0.1187
2021	2,087	0.0864
2022	5,050	0.2091
2023	4,856	0.201
2024	2,221	0.0919
**Outcome**		
CA	13	0
DE	1,379	0.048
DS	119	0.004
HO	6,034	0.21
LT	179	0.006
OT	9,784	0.34
RI	16	0.001
Not specified	11,269	0.391
**Time to onset (days)**		
0–30	708	0.185
31–60	296	0.077
61–90	217	0.057
91–180	410	0.107
181–360	527	0.138
>360	1,669	0.436

AEs, adverse events; US, United States; HO, hospitalization; LT, life-threatening; DS, disability; RI, required intervention; DE, death; OT, other outcomes.

### Disproportionality analysis of SOC levels

**Figure 2:**
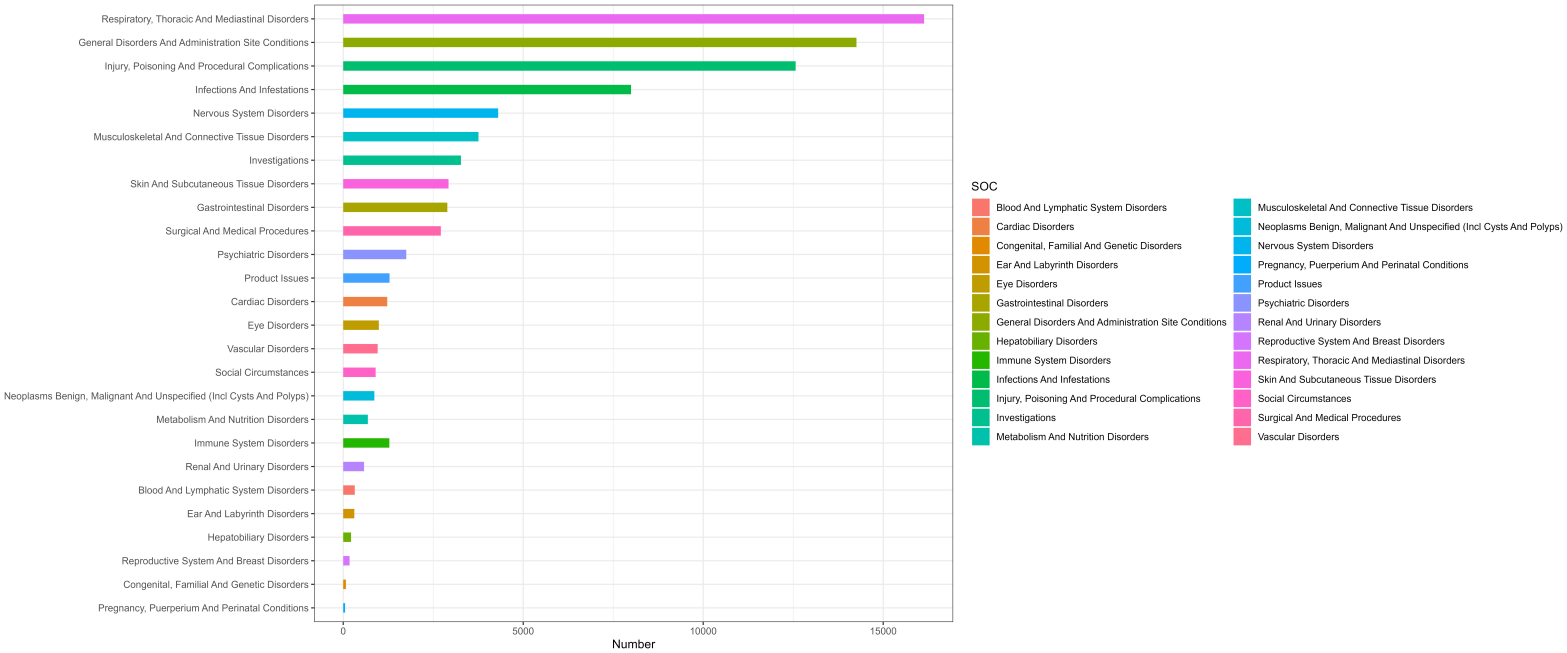
System organ classes distribution of mepolizumab-related AEs. SOC, system organ classes; AEs, adverse events.

In our analysis, we identified mepolizumab-associated AE signals across all 27 SOCs, as detailed in Table 2 and illustrated in Figure 2. Notably, several SOCs were flagged as significant based on the ROR-positive signaling criteria, which included respiratory, thoracic, and mediastinal disorders, with a substantial ROR of 5.12 (95% CI 5.03–5.21), suggesting a notably higher incidence of AEs in these systems. Additionally, social circumstances were signaled with an ROR of 2.43 (95% CI 2.27–2.59), and surgical and medical procedures were also marked by an elevated ROR of 2.41 (95% CI 2.32–2.50). Other notable findings included infections and infestations with an ROR of 1.86 (95% CI 1.81–1.90); injury, poisoning, and procedural complications with an ROR of 1.35 (95% CI 1.32–1.37); and immune system disorders with an ROR of 1.14 (95% CI 1.08–1.21). These six SOC signaling findings highlight the specific organ systems in which mepolizumab-induced AEs were most frequently reported, highlighting critical areas that warrant further attention and detailed investigation, emphasizing the need for targeted monitoring and potential revisions to patient management strategies.

**Table 2:** Disproportionality analysis of mepolizumab-related AEs in SOC level.

**SOC**	**a**	**b**	**c**	**d**	**ROR (95% CI)**	**PRR (χ2)**	**EBGM (Lower limit of the 95% CI)**
Respiratory, thoracic and mediastinal disorders	16,136	66,342	1,623,032	34,149,902	5.12 (5.03–5.21)	4.31 (42,592.6)	4.28 (4.22)
Social circumstances	900	81,578	161,743	35,611,191	2.43 (2.27–2.59)	2.41 (744.25)	2.41 (2.28)
Surgical and medical procedures	2,712	79,766	498,195	35,274,739	2.41 (2.32–2.5)	2.36 (2,146.29)	2.35 (2.28)
Infections and infestations	7,995	74,483	1,955,341	33,817,593	1.86 (1.81–1.9)	1.77 (2,841.35)	1.77 (1.74)
Injury, poisoning and procedural complications	12,568	69,910	4,208,192	31,564,742	1.35 (1.32–1.37)	1.3 (956.42)	1.29 (1.27)
Immune system disorders	1,136	81,342	432,311	35,340,623	1.14 (1.08–1.21)	1.14 (19.64)	1.14 (1.08)
General disorders and administration site conditions	14,257	68,221	6,371,543	29,401,391	0.96 (0.95–0.98)	0.97 (15.51)	0.97 (0.96)
Product issues	1,287	81,191	630,502	35,142,432	0.88 (0.84–0.93)	0.89 (19.42)	0.89 (0.85)
Musculoskeletal and connective tissue disorders	3,757	78,721	1,858,061	33,914,873	0.87 (0.84–0.9)	0.88 (68.23)	0.88 (0.85)
Ear and labyrinth disorders	308	82,170	156,321	35,616,613	0.85 (0.76–0.96)	0.85 (7.64)	0.85 (0.78)
Cardiac disorders	1,222	81,256	729,526	35,043,408	0.72 (0.68–0.76)	0.73 (128.2)	0.73 (0.69)
Investigations	3,270	79,208	2,084,922	33,688,012	0.67 (0.64–0.69)	0.68 (521.01)	0.68 (0.66)
Endocrine disorders	145	82,333	94,104	35,678,830	0.67 (0.57–0.79)	0.67 (23.9)	0.67 (0.58)
Nervous system disorders	4,304	78,174	2,773,592	32,999,342	0.66 (0.64–0.68)	0.67 (739.85)	0.67 (0.66)
Skin and subcutaneous tissue disorders	2,925	79,553	2,035,958	33,736,976	0.61 (0.59–0.63)	0.62 (705.92)	0.62 (0.6)
Eye disorders	989	81,489	694,944	35,077,990	0.61 (0.58–0.65)	0.62 (239.03)	0.62 (0.59)
Vascular disorders	957	81,521	687,041	35,085,893	0.6 (0.56–0.64)	0.6 (252.72)	0.6 (0.57)
Metabolism and nutrition disorders	684	81,794	723,365	35,049,569	0.41 (0.38–0.44)	0.41 (591.71)	0.41 (0.39)
Gastrointestinal disorders	2,892	79,586	2,966,487	32,806,447	0.4 (0.39–0.42)	0.42 (2,481.67)	0.42 (0.41)
Psychiatric disorders	1,751	80,727	1,909,836	33,863,098	0.38 (0.37–0.4)	0.4 (1,686.03)	0.4 (0.38)
Renal and urinary disorders	578	81,900	698,752	35,074,182	0.35 (0.33–0.38)	0.36 (675.03)	0.36 (0.34)
Neoplasms benign, malignant and unspecified (incl cysts and polyps)	865	81,613	1,095,325	34,677,609	0.34 (0.31–0.36)	0.34 (1,125.2)	0.34 (0.32)
Hepatobiliary disorders	217	82,261	291,581	35,481,353	0.32 (0.28–0.37)	0.32 (310.61)	0.32 (0.29)
Congenital, familial and genetic disorders	74	82,404	99,726	35,673,208	0.32 (0.26–0.4)	0.32 (105.96)	0.32 (0.27)
Reproductive system and breast disorders	176	82,302	268,293	35,504,641	0.28 (0.24–0.33)	0.28 (318.83)	0.28 (0.25)
Blood and lymphatic system disorders	321	82,157	586,024	35,186,910	0.23 (0.21–0.26)	0.24 (798.02)	0.24 (0.22)
Pregnancy, puerperium and perinatal conditions	52	82,426	138,054	35,634,880	0.16 (0.12–0.21)	0.16 (223.57)	0.16 (0.13)

AEs, adverse events; SOC, system organ classes; ROR, reporting odds ratio; PRR, proportional reporting ratio; EBGM, empirical Bayesian geometric mean; CI, confidence interval.

### Disproportionality analysis of PTs level

**Figure 3:**
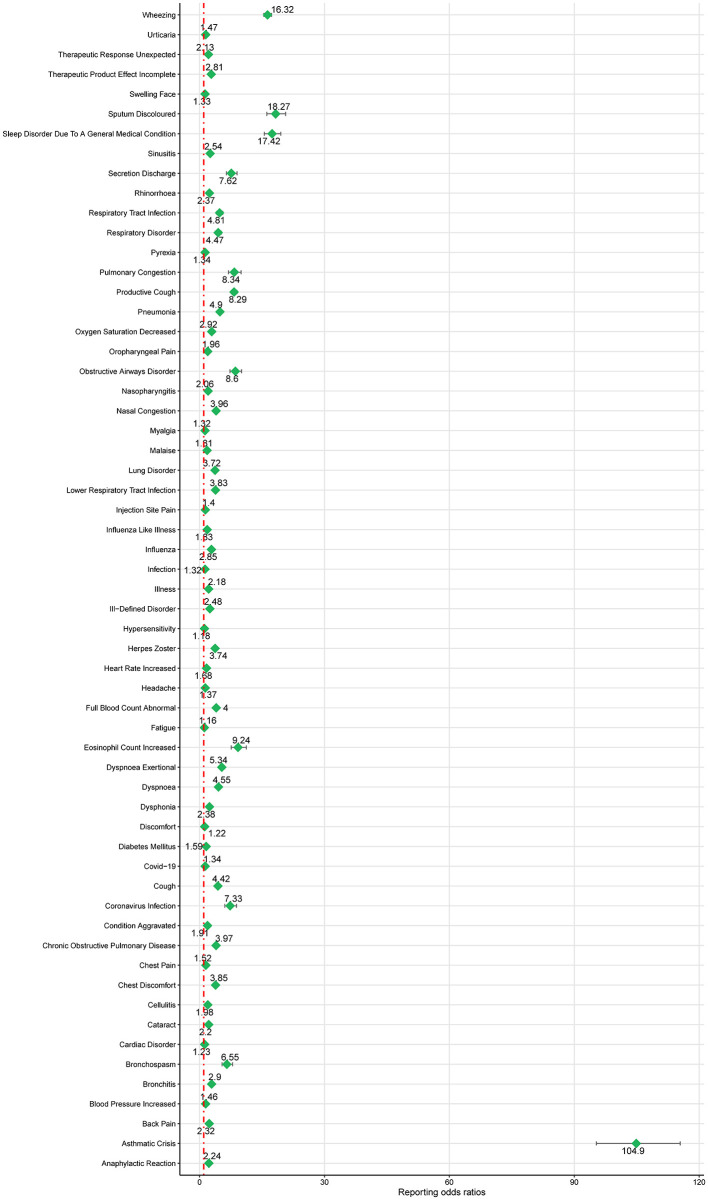
Forest plot of ROR for mepolizumab-associated AEs (a ≥ 100). AEs, adverse events; ROR, reporting odds ratio.

**Figure 4:**
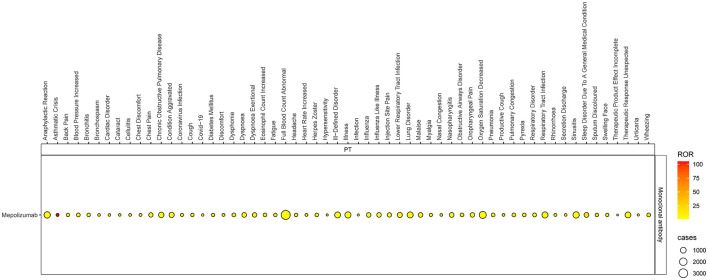
Heatmap of ROR for mepolizumab-associated AEs (a ≥ 100). AEs, adverse events.

Based on the SOC and adherence to the ROR signal criterion, we conducted a deeper analysis of significant PT signals within each SOC stratum that reported more than 100 cases (Table 3). Within the infections and infestations category, the three most significant PT signals identified were coronavirus infection (ROR = 7.33, 95% CI 6.05–8.88), pneumonia (ROR = 4.90, 95% CI 4.69–5.12), and respiratory tract infection (ROR = 4.81, 95% CI 4.14–5.59). Among the SOC of investigations, the strongest PT signal was observed for eosinophil count increased (ROR = 9.24, 95% CI 7.6–11.23). In the respiratory, thoracic, and mediastinal diseases category, particularly alarming were the incidences of asthma crises (ROR = 104.9, 95% CI 95.31–115.44) and wheezing (ROR = 16.32, 95% CI 15.42–17.28). In the psychiatric disorders category, the most significant finding was sleep disorders (ROR = 17.42, 95% CI 15.55–19.51). Additionally, within the immune system disorders, anaphylactic reaction was notably significant (ROR = 2.24, 95% CI 1.91–2.63). The comprehensive details of these findings are presented in Table 3 and Figures 3, 4, illustrating the scope and specificity of the AE signals linked to mepolizumab.

**Table 3:** Disproportionality analysis of mepolizumab-related AEs in PT level (a ≥ 100).

**PT**	**a**	**b**	**c**	**d**	**SOC**	**ROR (95% CI)**	**PRR (χ2)**	**EBGM (Lower limit of the 95% CI)**
Diabetes mellitus	135	82,343	36,962	35,735,972	Metabolism and nutrition disorders	1.59 (1.34–1.88)	1.58 (29)	1.58 (1.37)
Coronavirus infection	107	82,371	6,338	35,766,596	Infections and infestations	7.33 (6.05–8.88)	7.32 (574.51)	7.22 (6.15)
Pneumonia	2,141	80,337	193,520	35,579,414	Infections and infestations	4.9 (4.69–5.12)	4.8 (6402.3)	4.76 (4.59)
Respiratory tract infection	173	82,305	15,632	35,757,302	Infections and infestations	4.81 (4.14–5.59)	4.8 (514.98)	4.76 (4.2)
Lower respiratory tract infection	252	82,226	28,639	35,744,295	Infections and infestations	3.83 (3.38–4.33)	3.82 (519.62)	3.79 (3.42)
Herpes zoster	299	82,179	34,811	35,738,123	Infections and infestations	3.74 (3.33–4.19)	3.73 (591.65)	3.7 (3.37)
Influenza	450	82,028	68,755	35,704,179	Infections and infestations	2.85 (2.6–3.13)	2.84 (533.5)	2.83 (2.62)
Bronchitis	283	82,195	42,388	35,730,546	Infections and infestations	2.9 (2.58–3.26)	2.9 (349.31)	2.88 (2.61)
Sinusitis	357	82,121	61,159	35,711,775	Infections and infestations	2.54 (2.29–2.82)	2.53 (329.5)	2.52 (2.31)
Nasopharyngitis	538	81,940	113,561	35,659,373	Infections and infestations	2.06 (1.89–2.24)	2.05 (290.86)	2.05 (1.91)
Cellulitis	130	82,348	28,568	35,744,366	Infections and infestations	1.98 (1.66–2.35)	1.97 (62.21)	1.97 (1.7)
COVID-19	483	81,995	156,089	35,616,845	Infections and infestations	1.34 (1.23–1.47)	1.34 (42.18)	1.34 (1.24)
Infection	262	82,216	86,317	35,686,617	Infections and infestations	1.32 (1.17–1.49)	1.32 (19.92)	1.32 (1.19)
Eosinophil count increased	103	82,375	4,840	35,768,094	Investigations	9.24 (7.6–11.23)	9.23 (740.22)	9.06 (7.69)
Full blood count abnormal	183	82,295	19,886	35,753,048	Investigations	4 (3.46–4.63)	3.99 (406.75)	3.96 (3.51)
Oxygen saturation decreased	227	82,251	33,772	35,739,162	Investigations	2.92 (2.56–3.33)	2.92 (284)	2.9 (2.6)
Heart rate increased	206	82,272	53,150	35,719,784	Investigations	1.68 (1.47–1.93)	1.68 (56.7)	1.68 (1.5)
Blood pressure increased	303	82,175	90,291	35,682,643	Investigations	1.46 (1.3–1.63)	1.46 (43.16)	1.45 (1.32)
Headache	1,141	81,337	361,852	35,411,082	Nervous system disorders	1.37 (1.29–1.46)	1.37 (113.56)	1.37 (1.3)
Back pain	708	81,770	133,099	35,639,835	Musculoskeletal and connective tissue disorders	2.32 (2.15–2.5)	2.31 (523.51)	2.3 (2.16)
Myalgia	276	82,202	90,819	35,682,115	Musculoskeletal and connective tissue disorders	1.32 (1.17–1.48)	1.32 (21.18)	1.32 (1.19)
Asthmatic crisis	522	81,956	2,172	35,770,762	Respiratory, thoracic and mediastinal disorders	104.9 (95.31–115.44)	104.24 (43,034.77)	84.23 (77.75)
Sputum discolored	261	82,217	6,213	35,766,721	Respiratory, thoracic and mediastinal disorders	18.27 (16.14–20.69)	18.22 (4,077.31)	17.53 (15.8)
Wheezing	1,242	81,236	33,472	35,739,462	Respiratory, thoracic and mediastinal disorders	16.32 (15.42–17.28)	16.09 (16,968.96)	15.55 (14.83)
Productive cough	559	81,919	29,433	35,743,501	Respiratory, thoracic and mediastinal disorders	8.29 (7.62–9.01)	8.24 (3,491.27)	8.1 (7.55)
Obstructive airways disorder	151	82,327	7,624	35,765,310	Respiratory, thoracic and mediastinal disorders	8.6 (7.32–10.11)	8.59 (993.27)	8.44 (7.38)
Pulmonary congestion	121	82,357	6,303	35,766,631	Respiratory, thoracic and mediastinal disorders	8.34 (6.96–9.98)	8.33 (765.47)	8.19 (7.04)
Bronchospasm	104	82,374	6,892	35,766,042	Respiratory, thoracic and mediastinal disorders	6.55 (5.4–7.95)	6.54 (481.39)	6.46 (5.5)
Dyspnoea exertional	280	82,198	22,786	35,750,148	Respiratory, thoracic and mediastinal disorders	5.34 (4.75–6.01)	5.33 (973.53)	5.28 (4.78)
Dyspnoea	3,185	79,293	313,136	35,459,798	Respiratory, thoracic and mediastinal disorders	4.55 (4.39–4.71)	4.41 (8,392.24)	4.38 (4.25)
Cough	1,688	80,790	168,314	35,604,620	Respiratory, thoracic and mediastinal disorders	4.42 (4.21–4.64)	4.35 (4,331.82)	4.32 (4.15)
Respiratory disorder	164	82,314	15,928	35,757,006	Respiratory, thoracic and mediastinal disorders	4.47 (3.83–5.22)	4.47 (436.81)	4.43 (3.89)
Nasal congestion	314	82,164	34,452	35,738,482	Respiratory, thoracic and mediastinal disorders	3.96 (3.55–4.43)	3.95 (687.1)	3.93 (3.58)
Chronic obstructive pulmonary disease	253	82,225	27,682	35,745,252	Respiratory, thoracic and mediastinal disorders	3.97 (3.51–4.5)	3.96 (556.08)	3.94 (3.55)
Lung disorder	237	82,241	27,666	35,745,268	Respiratory, thoracic and mediastinal disorders	3.72 (3.28–4.23)	3.72 (466.73)	3.69 (3.32)
Rhinorrhoea	221	82,257	40,498	35,732,436	Respiratory, thoracic and mediastinal disorders	2.37 (2.08–2.71)	2.37 (173.7)	2.36 (2.11)
Dysphonia	188	82,290	34,258	35,738,676	Respiratory, thoracic and mediastinal disorders	2.38 (2.06–2.75)	2.38 (149.78)	2.37 (2.1)
Oropharyngeal pain	254	82,224	56,234	35,716,700	Respiratory, thoracic and mediastinal disorders	1.96 (1.73–2.22)	1.96 (118.91)	1.95 (1.76)
Sleep disorder due to a general medical condition	311	82,167	7,772	35,765,162	Psychiatric disorders	17.42 (15.55–19.51)	17.36 (4,610.17)	16.73 (15.21)
Anaphylactic reaction	152	82,326	29,474	35,743,460	Immune system disorders	2.24 (1.91–2.63)	2.24 (103.5)	2.23 (1.95)
Hypersensitivity	300	82,178	110,697	35,662,237	Immune system disorders	1.18 (1.05–1.32)	1.18 (7.86)	1.17 (1.07)
Urticaria	306	82,172	90,597	35,682,337	Skin and subcutaneous tissue disorders	1.47 (1.31–1.64)	1.46 (45.12)	1.46 (1.33)
Secretion discharge	140	82,338	7,983	35,764,951	General disorders and administration site conditions	7.62 (6.44–9)	7.61 (789.63)	7.49 (6.51)
Chest discomfort	486	81,992	54,946	35,717,988	General disorders and administration site conditions	3.85 (3.52–4.21)	3.84 (1,011.77)	3.81 (3.54)
Therapeutic product effect incomplete	552	81,926	85,511	35,687,423	General disorders and administration site conditions	2.81 (2.59–3.06)	2.8 (636.1)	2.79 (2.6)
Ill-defined disorder	241	82,237	42,202	35,730,732	General disorders and administration site conditions	2.48 (2.19–2.82)	2.48 (211.27)	2.47 (2.22)
Illness	333	82,145	66,341	35,706,593	General disorders and administration site conditions	2.18 (1.96–2.43)	2.18 (211.26)	2.17 (1.98)
Therapeutic response unexpected	144	82,334	29,367	35,743,567	General disorders and administration site conditions	2.13 (1.81–2.51)	2.13 (85.61)	2.12 (1.85)
Condition aggravated	843	81,635	192,677	35,580,257	General disorders and administration site conditions	1.91 (1.78–2.04)	1.9 (358.32)	1.89 (1.79)
Malaise	1,099	81,379	264,616	35,508,318	General disorders and administration site conditions	1.81 (1.71–1.92)	1.8 (393.07)	1.8 (1.71)
Influenza like illness	175	82,303	41,475	35,731,459	General disorders and administration site conditions	1.83 (1.58–2.13)	1.83 (65.69)	1.83 (1.61)
Chest pain	312	82,166	89,408	35,683,526	General disorders and administration site conditions	1.52 (1.36–1.69)	1.51 (54.31)	1.51 (1.38)
Injection site pain	502	81,976	155,982	35,616,952	General disorders and administration site conditions	1.4 (1.28–1.53)	1.4 (56.43)	1.39 (1.3)
Pyrexia	589	81,889	190,844	35,582,090	General disorders and administration site conditions	1.34 (1.24–1.45)	1.34 (50.56)	1.34 (1.25)
Fatigue	1,279	81,199	478,883	35,294,051	General disorders and administration site conditions	1.16 (1.1–1.23)	1.16 (28)	1.16 (1.11)
Swelling face	105	82,373	34,199	35,738,735	General disorders and administration site conditions	1.33 (1.1–1.61)	1.33 (8.65)	1.33 (1.13)
Discomfort	107	82,371	38,116	35,734,818	General disorders and administration site conditions	1.22 (1.01–1.47)	1.22 (4.15)	1.22 (1.04)
Cardiac disorder	133	82,345	46,857	35,726,077	Cardiac disorders	1.23 (1.04–1.46)	1.23 (5.76)	1.23 (1.07)
Cataract	172	82,306	34,004	35,738,930	Eye disorders	2.2 (1.89–2.55)	2.19 (111.29)	2.19 (1.93)

AEs, adverse events; PT, preferred term; ROR, reporting odds ratio; PRR, proportional reporting ratio; EBGM, empirical Bayesian geometric mean; CI, confidence interval.

### Subgroup analysis

**Figure 5:**
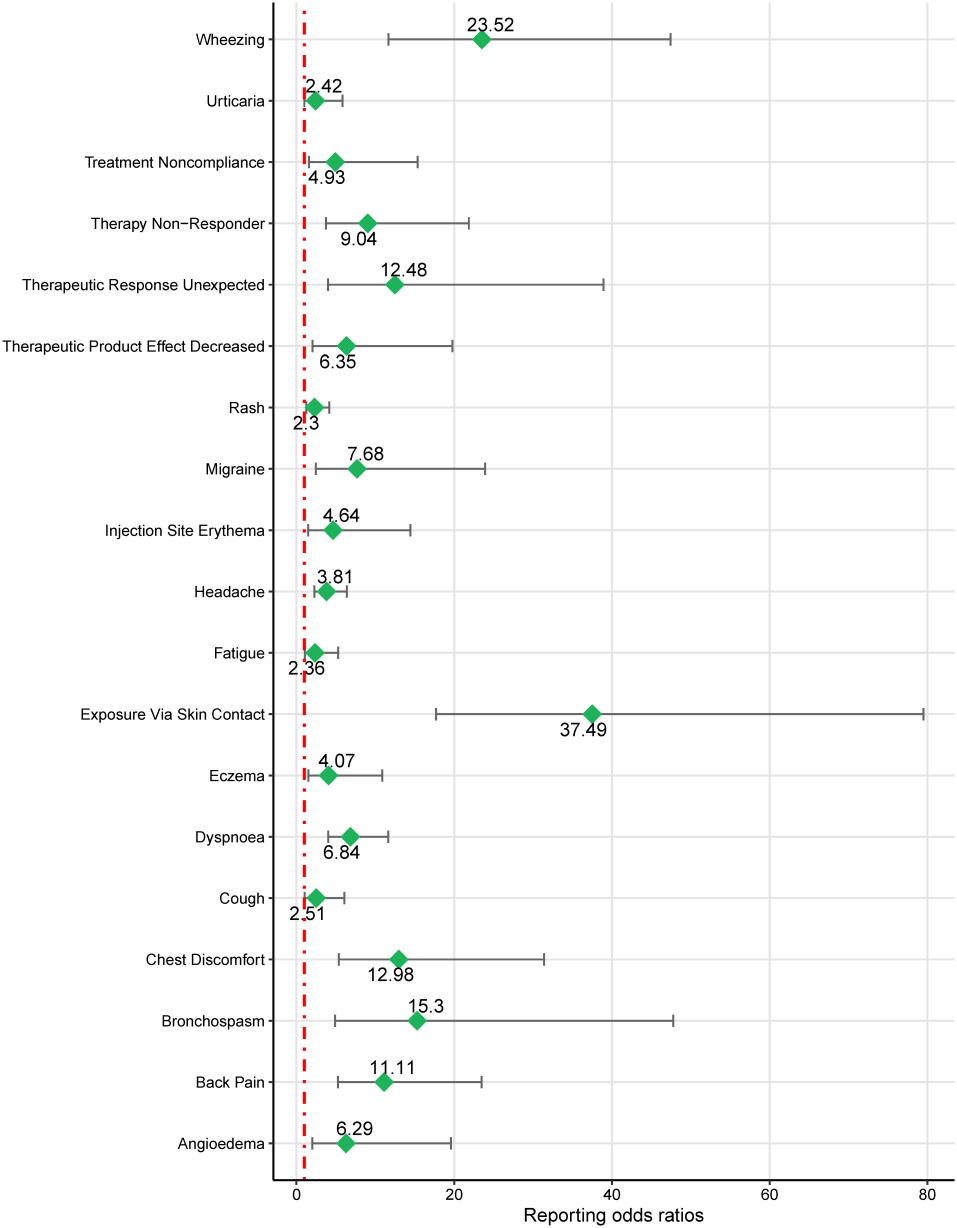
Forest plot of ROR for mepolizumab-associated AEs in people < 18 years old (a ≥ 3). AEs, adverse events; ROR, reporting odds ratio.

Considering the diverse indications for mepolizumab, we conducted a subgroup analysis of patients aged < 18 years old. Four significant SOC signals were associated with mepolizumab use (Table 4) were identified based on the ROR-positive signaling criteria, indicating a substantial correlation with AEs in this age group. Respiratory, thoracic, and mediastinal disorders were the most noteworthy, with a high ROR of 5.48 (95% CI 4.17–6.68). Ear and labyrinthine disorders also showed significant signals with an ROR of 3.44 (95% CI 1.29–9.21), followed by general disorders and administration site conditions (ROR = 1.49, 95% CI 1.19–1.88), and injury, poisoning, and procedural complications (ROR = 1.26, 95% CI 1.00–1.58). These results highlight the specific organ systems in which mepolizumab-induced AEs are most frequently reported in individuals under 18 years of age. Notable PT results within these SOC strata included wheezing, bronchospasms, and chest discomfort. Further details of these PT findings are shown in Figure 5 and outlined in Table 5, which provides a comprehensive view of mepolizumab-associated risk in this demographic.

**Table 4:** Disproportionality analysis of mepolizumab-associated AEs at SOC level in people < 18 years old.

**SOC**	**a**	**b**	**c**	**d**	**ROR (95% CI)**	**PRR (χ2)**	**EBGM (Lower limit of the 95% CI)**
Musculoskeletal and connective tissue disorders	18	454	31,125	1,239,644	1.58 (0.99–2.53)	1.56 (3.67)	1.56 (1.05)
General disorders and administration site conditions	89	383	170,996	1,099,773	1.49 (1.19–1.88)	1.4 (11.81)	1.4 (1.16)
Nervous system disorders	31	441	95,225	1,175,544	0.87 (0.6–1.25)	0.88 (0.58)	0.88 (0.65)
Gastrointestinal disorders	21	451	87,685	1,183,084	0.63 (0.41–0.97)	0.64 (4.41)	0.64 (0.45)
Vascular disorders	3	469	20,690	1,250,079	0.39 (0.12–1.2)	0.39 (2.9)	0.39 (0.15)
Respiratory, thoracic and mediastinal disorders	84	388	50,061	1,220,708	5.28 (4.17–6.68)	4.52 (239.12)	4.51 (3.70)
Injury, poisoning and procedural complications	92	380	205,319	1,065,450	1.26 (1–1.58)	1.21 (3.87)	1.21 (1)
Psychiatric disorders	10	462	82,293	1,188,476	0.31 (0.17–0.58)	0.33 (14.79)	0.33 (0.19)
Infections and infestations	22	450	74,848	1,195,921	0.78 (0.51–1.2)	0.79 (1.29)	0.79 (0.55)
Skin and subcutaneous tissue disorders	36	436	145,798	1,124,971	0.64 (0.45–0.9)	0.66 (6.87)	0.66 (0.5)
Neoplasms benign, malignant and unspecified (incl cysts and polyps)	1	471	11,140	1,259,629	0.24 (0.03–1.71)	0.24 (2.4)	0.24 (0.05)
Immune system disorders	8	464	18,952	1,251,817	1.14 (0.57–2.29)	1.14 (0.13)	1.14 (0.63)
Investigations	13	459	66,326	1,204,443	0.51 (0.3–0.89)	0.53 (5.8)	0.53 (0.33)
Surgical and medical procedures	4	468	11,034	1,259,735	0.98 (0.36–2.61)	0.98 (0)	0.98 (0.43)
Blood and lymphatic system disorders	5	467	31,300	1,239,469	0.42 (0.18–1.02)	0.43 (3.87)	0.43 (0.21)
Ear and labyrinth disorders	4	468	3,148	1,267,621	3.44 (1.29–9.21)	3.42 (6.86)	3.42 (1.5)
Renal and urinary disorders	2	470	15,864	1,254,905	0.34 (0.08–1.35)	0.34 (2.6)	0.34 (0.11)
Eye disorders	12	460	21,074	1,249,695	1.55 (0.87–2.74)	1.53 (2.26)	1.53 (0.95)
Cardiac disorders	2	470	20,573	1,250,196	0.26 (0.06–1.04)	0.26 (4.23)	0.26 (0.08)
Metabolism and nutrition disorders	1	471	26,403	1,244,366	0.1 (0.01–0.71)	0.1 (8.08)	0.1 (0.02)
Social circumstances	2	470	3,515	1,267,254	1.53 (0.38–6.15)	1.53 (0.37)	1.53 (0.48)
Reproductive system and breast disorders	1	471	15,498	1,255,271	0.17 (0.02–1.22)	0.17 (3.98)	0.17 (0.03)
Product issues	9	463	38,664	1,232,105	0.62 (0.32–1.2)	0.63 (2.06)	0.63 (0.36)
Endocrine disorders	1	471	4,414	1,266,355	0.61 (0.09–4.33)	0.61 (0.25)	0.61 (0.12)
Hepatobiliary disorders	1	471	11,995	1,258,774	0.22 (0.03–1.59)	0.22 (2.71)	0.22 (0.04)

AEs, adverse events; SOC, system organ classes; ROR, reporting odds ratio; PRR, proportional reporting ratio; EBGM, empirical Bayesian geometric mean; CI, confidence interval.

**Table 5:** Disproportionality analysis of mepolizumab-related AEs at PT level in people < 18 years old (a ≥ 3).

**PT**	**a**	**b**	**c**	**d**	**SOC**	**ROR (95% CI)**	**PRR (χ2)**	**EBGM (Lower limit of the 95% CI)**
Headache	15	457	10,840	1,259,929	Nervous system disorders	3.81 (2.28–6.38)	3.73 (30.12)	3.72 (2.42)
Dyspnoea	14	458	5,651	1,265,118	Respiratory, thoracic and mediastinal disorders	6.84 (4.02–11.65)	6.67 (67.61)	6.66 (4.26)
Rash	11	461	13,076	1,257,693	Skin and subcutaneous tissue disorders	2.3 (1.26–4.17)	2.26 (7.84)	2.26 (1.37)
Wheezing	8	464	931	1,269,838	Respiratory, thoracic and mediastinal disorders	23.52 (11.66–47.44)	23.13 (168.1)	22.95 (12.75)
Back pain	7	465	1,719	1,269,050	Musculoskeletal and connective tissue disorders	11.11 (5.26–23.48)	10.96 (63.21)	10.92 (5.84)
Exposure via skin contact	7	465	510	1,270,259	Injury, poisoning and procedural complications	37.49 (17.69–79.49)	36.95 (241.65)	36.47 (19.45)
Fatigue	6	466	6,890	1,263,879	General disorders and administration site conditions	2.36 (1.06–5.29)	2.34 (4.65)	2.34 (1.19)
Chest discomfort	5	467	1,047	1,269,722	General disorders and administration site conditions	12.98 (5.37–31.41)	12.86 (54.46)	12.8 (6.11)
Cough	5	467	5,390	1,265,379	Respiratory, thoracic and mediastinal disorders	2.51 (1.04–6.07)	2.5 (4.5)	2.5 (1.19)
Urticaria	5	467	5,589	1,265,180	Skin and subcutaneous tissue disorders	2.42 (1–5.85)	2.41 (4.13)	2.41 (1.15)
Therapy non-responder	5	467	1,503	1,269,266	General disorders and administration site conditions	9.04 (3.74–21.86)	8.96 (35.27)	8.93 (4.27)
Eczema	4	468	2,665	1,268,104	Skin and subcutaneous tissue disorders	4.07 (1.52–10.89)	4.04 (9.16)	4.04 (1.77)
Therapeutic response unexpected	3	469	651	1,270,118	General disorders and administration site conditions	12.48 (4–38.94)	12.41 (31.33)	12.35 (4.77)
Therapeutic product effect decreased	3	469	1,279	1,269,490	General disorders and administration site conditions	6.35 (2.04–19.78)	6.32 (13.4)	6.3 (2.44)
Migraine	3	469	1,058	1,269,711	Nervous system disorders	7.68 (2.46–23.93)	7.63 (17.26)	7.62 (2.94)
Bronchospasm	3	469	531	1,270,238	Respiratory, thoracic and mediastinal disorders	15.3 (4.9–47.77)	15.21 (39.62)	15.13 (5.84)
Treatment non-compliance	3	469	1,646	1,269,123	General disorders and administration site conditions	4.93 (1.58–15.36)	4.91 (9.33)	4.9 (1.89)
Angioedema	3	469	1,291	1,269,478	Skin and subcutaneous tissue disorders	6.29 (2.02–19.6)	6.26 (13.23)	6.24 (2.41)
Injection site erythema	3	469	1,750	1,269,019	General disorders and administration site conditions	4.64 (1.49–14.45)	4.62 (8.49)	4.61 (1.78)

AEs, adverse events; PT, preferred term; ROR, reporting odds ratio; PRR, proportional reporting ratio; EBGM, empirical Bayesian geometric mean; CI, confidence interval.

### TTO and WSP analysis

**Figure 6:**
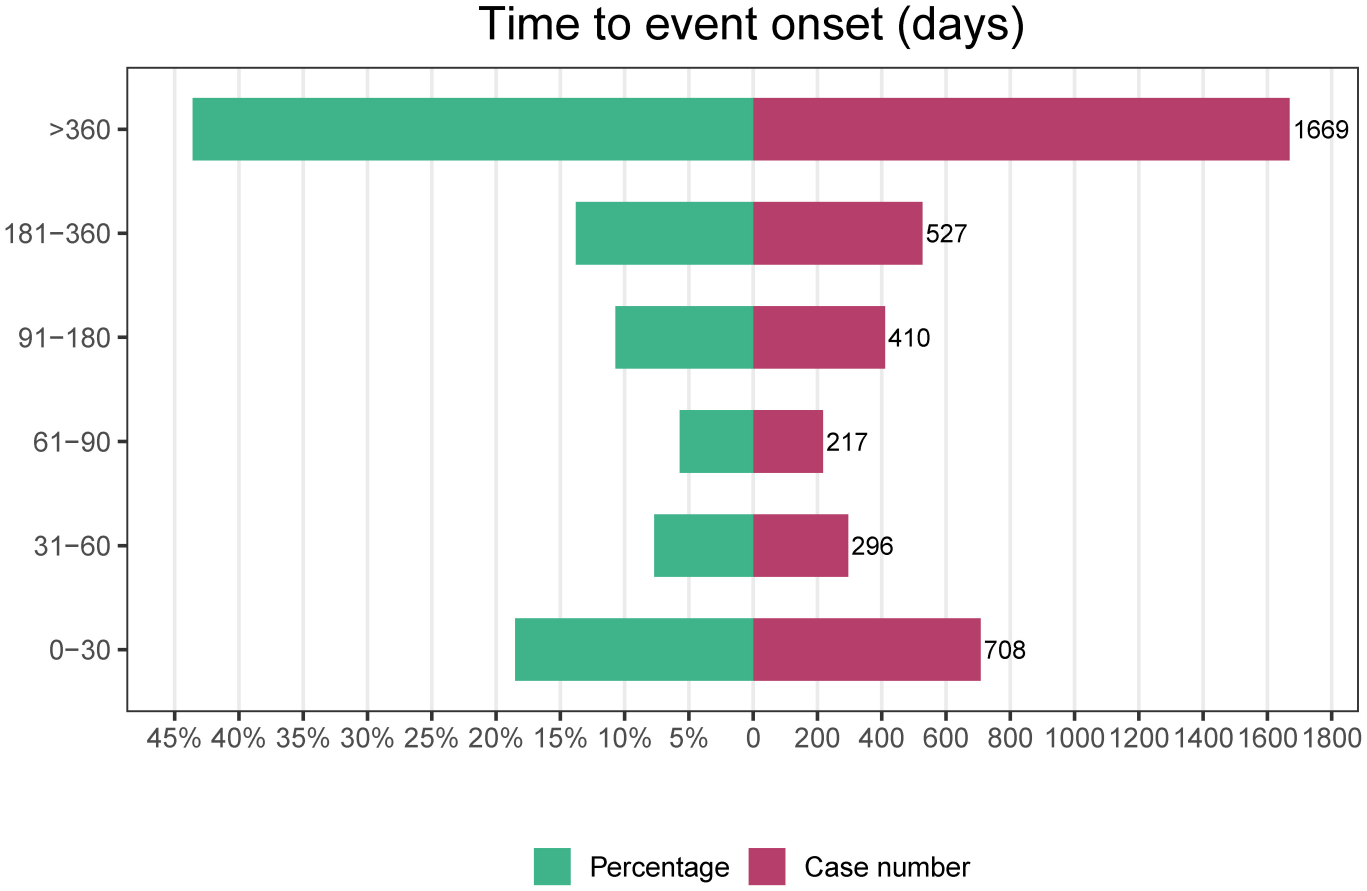
Time-to-onset analysis of mepolizumab-related AEs. AEs, adverse events.

Figure 6 provides a comprehensive visual representation of the TTO analysis of all AEs associated with mepolizumab use. This figure illustrates the temporal distribution of these events, offering valuable insights into the timing of post-treatment initiation.

Building on this, our subsequent analysis using the WSP revealed critical insights. Both the shape parameter (β) and the upper limit of its 95% CI were below 1, as detailed in Table 6. This finding indicates a trend toward an “early failure-type” pattern for mepolizumab-related AEs, suggesting that these AEs are more likely to occur soon after treatment initiation rather than at later stages of the treatment course. These findings emphasize the need for increased monitoring and precautions during the initial stages of mepolizumab therapy.

**Table 6:** Time-to-onset and WSP analysis of mepolizumab-induced AEs.

**Weibull distribution**		
**Scale parameter**	**Shape parameter**	**Failure type**
α (95% CI)	β (95% CI)	Early failure
415.47 (395.67–435.27)	0.70 (0.68–0.72)	

AEs, adverse events; WSP, Weibull Shape Parameter; CI, confidence interval.

## Discussion

In this study, we conducted a comprehensive exploration of the safety profile of mepolizumab-use based on the FAERS database. Several new AEs associated with mepolizumab treatment were identified, including pneumonitis, wheezing, vocal difficulties, and sleep disturbances. Additionally, both the long-term safety of mepolizumab and its specific safety considerations in patients under 18 years of age were evaluated. To the best of our knowledge, this represents the most recent pharmacovigilance analysis focused on the post-marketing safety of mepolizumab. The insights gained from this study provide a valuable resource for further refinement of clinical management and utilization of mepolizumab to enhance treatment strategies and improve patient outcomes.

Mepolizumab, an IL-5 antagonist monoclonal antibody, targets IL-5, a crucial cytokine involved in eosinophil growth, differentiation, aggregation, activation, and survival. It binds to IL-5 and inhibits its biological activity, preventing IL-5 from interacting with the α chain of the IL-5 receptor complex on the surface of eosinophils. With the increasing use of mepolizumab, there has been a corresponding increase in the number of documented AEs, underscoring the need for ongoing surveillance of these events. Our findings revealed significant positive signals at the SOC level in six categories: respiratory, thoracic, and mediastinal disorders; social circumstances; surgical and medical procedures; infections and infestations; injury, poisoning, and procedural complications; and immune system disorders. These results are not entirely consistent with those of two publications that analyzed AEs associated with mepolizumab (14, 15). However, as the number of AE reports has substantially increased, using the most recent AE data helps minimize the likelihood of false positives, affirming that respiratory, thoracic, and mediastinal disorders remain among the most critical classes of adverse reactions linked to mepolizumab use. Asthma crisis emerged as the strongest signal. While previous clinical trials consistently identified headache and nasopharyngitis as the most common AEs, asthma crisis was considered significant and serious (20–22). The COLUMBA study specifically identified asthma crisis as the most prevalent AE following long-term mepolizumab therapy (12). Similarly, the COSMEX study noted that asthma exacerbations ranked as the second most frequent AE after nasopharyngitis, particularly among patients with severe eosinophilic asthma (11). Importantly, patients with treatment intervals exceeding 12 weeks experienced deteriorating asthma symptoms, underscoring the risks associated with discontinuing monoclonal antibody therapy, which can exacerbate asthma symptoms. Additionally, our study identified other common AEs such as changes in sputum color, sleep disturbances, dyspnea, wheezing, and vocal difficulties. These findings highlight the importance of continued vigilance in monitoring the adverse reactions associated with mepolizumab use to enhance patient safety and treatment efficacy.

Moreover, mepolizumab use may be associated with an increased risk of infection. In a phase III clinical study by Pavord et al. mepolizumab was associated with an increased susceptibility to pneumonia in patients with eosinophilic chronic obstructive pulmonary disease (21). Our study found a correlation between mepolizumab use and upper and lower respiratory tract infections, influenza, sinusitis, nasopharyngitis, herpes zoster, cellulitis, and coronavirus disease 2019 (COVID-19) AEs. While some of these AEs are documented in the drug specifications, others, such as cellulitis and COVID-19, require further attention. Asthma itself does not increase the risk of severe acute respiratory syndrome coronavirus 2 (SARS-CoV-2) infection, and previous studies have suggested a negative association between asthma and COVID-19, possibly because of decreased angiotensin-converting enzyme 2 (ACE2) receptors found in patients with asthma (23, 24). We noted a correlation between mepolizumab and COVID-19, which may be attributed to the inhibition of IL-5 signaling by the drug and a reduction in Th2 responses. While mepolizumab reduces eosinophilia-related syndromes such as severe eosinophilic asthma, it may also suppress Th2 responses, which are crucial for viral immunity, thereby raising concerns about the risk of viral infections in patients treated with mepolizumab. The association of opportunistic infections, such as herpes zoster, with mepolizumab use is consistent with our results and supported by the findings of Khatri et al. (12). Additionally, Camiolo et al. observed increased ACE2 receptor expression in a subgroup of patients with asthma exhibiting elevated Th1 and reduced Th2 epithelial gene expression, suggesting that increased ACE2 receptor expression may exacerbate coronavirus-induced pneumonia, leading to potentially adverse outcomes and corroborating the potential for COVID-19 AEs (25–27). A recent study showed a significant decrease in eosinophil counts in patients treated with biologics, which was not associated with increased severity or mortality in COVID-19 pneumonia (28). In summary, mepolizumab, an anti-IL-5 monoclonal antibody used for severe eosinophilic asthma, may inadvertently increase the risk of opportunistic infections, including COVID-19, by altering the immune system dynamics. By targeting IL-5, mepolizumab effectively reduces eosinophil counts. Eosinophils are t only involved in allergic inflammation but also play a crucial role in antiviral defense, particularly in the respiratory tract. These cells contribute to the antiviral immunity by releasing cytotoxic granule proteins and promoting the recruitment of other immune cells. Consequently, their depletion can impair the body's ability to combat viral infections, potentially increasing the susceptibility to pathogens such as SARS-CoV-2. Furthermore, suppression of the Th2 immune response by mepolizumab may disrupt the balance between the Th1 and Th2 pathways. While beneficial for reducing eosinophilic inflammation, an imbalanced Th1/Th2 response can weaken the overall immune function, making patients more susceptible to opportunistic infections, such as herpes zoster. Alterations in immune response may also affect ACE2 receptor expression, potentially influencing the severity of COVID-19 in patients treated with mepolizumab. These findings have significant implications for clinical practice. Healthcare providers should monitor patients receiving mepolizumab for signs of infection, particularly respiratory and opportunistic infections. Preventive measures, including vaccination and patient education on infection risks, should be integral to management plans. Clinicians should reassess the risks and benefits of continuing mepolizumab therapy on a case-by-case basis during widespread viral outbreaks and pandemics. Future longitudinal studies are required to assess the long-term risk of infection associated with mepolizumab use in larger patient populations. Mechanistic studies exploring how mepolizumab affects the immune pathways involved in antiviral defense could provide deeper insights. Additionally, real-world data analyses can help translate these findings into practical guidelines aiming to optimize treatment regimens that control asthma while also minimizing the risk of infection. Such research is crucial for formulating strategies to mitigate potential AEs and ensure that the therapeutic benefits of mepolizumab are realized without compromising patient safety.

While leveraging real-world data mining techniques through the FAERS database offers significant benefits, it is essential to acknowledge the inherent limitations of pharmacovigilance databases and their potential impact on our findings. These limitations include false, underreported, inaccurate, incomplete, and delayed reports, which can introduce various biases into the analysis. Underreporting is common and may lead to an underestimation of the true incidence of AEs, whereas over-reporting of well-known AEs can skew the data toward certain outcomes. Moreover, the FAERS database lacks detailed information on the total number of patients exposed to mepolizumab and does not include a control group, making it impossible to calculate the true incidence rates of AEs specifically linked to the drug. This limitation affected our ability to generalize the findings and accurately assess the actual risks associated with mepolizumab use. Additionally, the nature of disproportionality analyses limits our ability to establish causal relationships between mepolizumab use and the observed AEs, as these analyses were designed to identify statistical associations rather than establish causation. The associations observed may be influenced by confounding factors such as the patients' underlying conditions, concomitant medications, or demographic characteristics that were not controlled for in the analysis. Residual confounding is also a concern, as unmeasured or unknown factors may affect the relationship between mepolizumab use and AEs. For example, patients treated with mepolizumab may have a more severe disease or a higher prevalence of comorbidities, which could independently increase the risk of certain AEs. Adjusting for all the potential confounders without access to comprehensive clinical data is challenging. These limitations may have affected the validity and reliability of our findings, highlighting the need for cautious interpretation. To strengthen the robustness and validity of our results, further research is imperative. This includes well-designed experimental studies, clinical trials, and observational studies, such as case-control or cohort studies, which can control for confounding variables and more accurately assess causality. Future studies should provide a more comprehensive understanding of the safety profile of mepolizumab and validate the associations observed in the current analysis.

## Conclusions

Our study underscores the critical role of post-marketing surveillance in evaluating the safety of mepolizumab using data from the FAERS database. We observed significant AE signals associated with mepolizumab use, particularly in the cases of respiratory diseases, infections, and immune system complications. We identified novel AE signals, such as COVID-19, that were previously underreported in drug documentation. These findings hold substantial clinical relevance, suggesting an elevated risk of certain opportunistic infections associated with mepolizumab use. The emergence of these novel AEs emphasizes the necessity for clinicians to remain vigilant in monitoring the signs of infection among patients treated with mepolizumab and to carefully weigh these potential risks when prescribing this medication. Furthermore, our results highlight the urgent need for continued research to confirm these associations and investigate the mechanisms underlying these AEs. This ongoing inquiry is essential to ensure optimal patient safety and provide insights for clinical guidelines.

## Data Availability

The original contributions presented in the study are included in the article/supplementary material, further inquiries can be directed to the corresponding authors.
